# Static Precision of Instrumented Pointers for Anatomical Landmark Calibration in CAST-Like Motion Analysis Measurements

**DOI:** 10.1007/s10439-025-03753-8

**Published:** 2025-05-09

**Authors:** Kristóf Rácz, Beáta Seregély, Rita M. Kiss

**Affiliations:** 1 Department of Mechatronics, Optics and Mechanical Engineering Informatics, Faculty of Mechanical Engineering, Budapest University of Technology and Economics, Műegyetem rkp. 3., H-1111 Budapest, Hungary; 2 Department of Physiotherapy, Faculty of Health Sciences, Semmelweis University, Vas utca 17., H-1088 Budapest, Hungary

**Keywords:** Calibrated anatomical systems technique, Motion analysis, Anatomical landmark, Instrumented pointer, Precision

## Abstract

**Purpose:**

The Calibrated Anatomical Systems Technique is an integral part of modern motion analysis systems. However, the calibration of anatomical landmarks is shown to have large variations in intra- and inter-examiner accuracy, which can result in both offset type errors or changes in the characteristics of joint angles and other parameters. This paper is the first instalment in a series of articles aiming to characterize and minimize all of the different factors contributing to these inconsistent calibrations by examining and optimizing the performance of the instrumented pointers used for landmark calibration. A complete characterisation of all aspects of instrumented pointer precision has not been done before.

**Methods:**

This paper focuses on examining four different pointers used with an optical OptiTrack motion capture system to establish the expected variability when measuring pointer tip location. Four different pointers were measured at three different locations within the motion capture system’s measurement volume, in a distinct orientation at each of these location.

**Results:**

A single stationary marker can be measured with less than 0.06 mm variation with 95% confidence, whilst the variation of the tip of a stationary pointer is 0.2 mm. If the pointer markers are located closer than what the motion capture system is able to resolve, these variations can more than double, but pointer geometry has limited effect on precision apart from this.

**Conclusion:**

Thanks to improvement of motion capture technology in the last 20 years, static precision is already excellent. Robustness of tracking can likely be improved, but it’s effect on overall pointer precision would be minimal and likely inconsequential.

## Introduction

Researchers use motion analysis to study and gain an understanding of biomechanical systems [1–5]. By measuring the movement of joint centres and other anatomical landmarks, the motion can be recreated on a simulated mechanical model [6–11] of the real system. This allows for the estimation and analysis of various parameters describing the motion, such as joint angles and spatio-temporal parameters. The smaller the deviation between the measured and the true instantaneous positions of the selected anatomical points throughout the motion, the more accurate the model’s results will be. However, there are several factors in the measurement process that can introduce errors into these recordings [12].

Cappozzo et al. introduced the Calibrated Anatomical Systems Technique (CAST) in 1995 [13]. It has since became a standard methodology for motion analysis studies based on motion capture (mocap) systems, due to its ability to considerably reduce soft-tissue artefacts that arise from the relative movement between the anatomical landmarks and the corresponding measured points on the skin surface during motion. Soft-tissue artefacts are considered to be the most relevant issue of accurate motion analysis [14]. With CAST, the studied person’s body segments are each tracked by a mocap system as 6 degrees of freedom rigid bodies using a cluster of markers. These markers are placed in a way as to minimise soft-tissue artefacts [15, 16], and are not limited by the location of the actual landmarks. The markers define a local coordinate system referred to as a technical frame, which is considered as though it is rigidly attached to the body segment’s bone (a bone-embedded frame). A calibration process which determines the relative (and fixed) position of the anatomical landmarks in their corresponding technical frames is required at the beginning of measurements. This calibration is performed by either attaching temporary markers on the selected anatomical landmarks, or by using an instrumented pointer. This latter option is preferable due to its lower ambiguity, as this pointer has a narrow pointer tip that can be used to mark the anatomical landmarks more precisely. The pointer also has several technical markers of its own used for tracking. The tip location is reconstructed based on its relative position compared to the pointer’s markers.

Similarly to soft-tissue artefacts that arise during the motion itself, the accuracy of calibrating these anatomical landmarks prior to the measurement can considerably impact the end results of the analysis. Despite being less frequently discussed than soft-tissue artefacts [17–20], a difference between the tracked and the actual landmark positions can introduce both offset errors and changes in the observed characteristics of joint kinematics [21–23], as well as changes in their kinetics such as joint moments [24]. As a result, accurate marking of the anatomical landmarks is essential for correct results.

The total error of the landmark calibration is the deviation between the location of the actual physical landmark and the reconstructed location of the pointer tip. Both of these can, and in practice will differ from the location of the physical pointer tip due to natural and metrological factors. Natural factors influence how accurately can the examiner locate a given anatomical landmark. Finding the landmarks rely on palpation, complicated by layers of soft tissues such as skin, fat and muscles, and variations in individual physiology [25]. The greater trochanter is a good example with a large surface and high individual variation in the volume of covering muscle and fat tissues, making it very difficult to mark precisely and consistently.

Metrological factors describe the accuracy of the examiner calibrating a precisely given physical location. This depends entirely on the capabilities of the examiner and the measurement system, and is separate from subject physiology. Metrological factors include several smaller aspects that build on each other. First is the mocap system’s accuracy of measuring individual marker positions, affected by noise and possibly systematic measurement error [26]. The individual noisy marker locations, and the trueness of the calibrated relative position of the tip with respect to the measured markers determine the reconstructed tip position in the measurement coordinate system. The examiner’s ability in placing the tip exactly at the intended point adds further error.

**Fig. 1:**
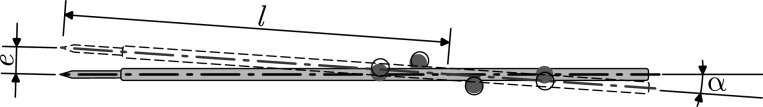
Effect of the pointer orientation error on the tip location. As a result of random noise in marker location, the physical tip of the pointer does not coincide with the measured location. Tip error is proportional to the length between the tip and marker barycentre: $e=l \cdot tan(\alpha )$. Position along the long axis of the pointer is affected much less than in the other two directions

The geometry of the pointer will determine robustness against marker measurement noise. A good marker arrangement can reduce the orientational and positional errors of the pointers technical frame by averaging and cancelling out the random components [27]. Orientational errors especially have a large influence on the reconstructed tip position through the “pivot” effect, where a small orientational error causes a larger displacement at the tip (Fig. 1).

Together, the natural and metrological factors introduce considerable error into the calibration process. Within- and between-examiner differences in the calibrated location of landmarks can be very large, often even 10 + millimetres [22, 28–31], which makes the reproduction and comparison of motion analysis studies difficult. Currently, natural factors are considered to be the main contributors, and efforts to reduce these (such as clear guidelines for identification and palpation of the landmarks [32, 33], and minimising soft-tissue artefacts [14, 17–20]) are ongoing and integral to the improvement of calibration accuracy.

On the other hand, metrological factors are usually considered insignificant, but to the authors knowledge, no previous study have attempted to verify this. On the contrary, Tawy and Rowe [34] found that purely instrumental errors from marking the same point with different pointer orientations could produce clinically meaningful kinematic errors of 5° due to incorrect determination of joint axes. No research have attempted to comprehensively examine and quantify the full range of metrological errors of motion capture-based instrumented pointers, or took a methodical approach to reduce them by improvement of the pointers and procedures.

Our goal is to characterise these metrological errors, understand their nature, find their causes and optimize what’s possible, preferably to the point where the performance of the pointer is truly an insignificant factor. *The aim of this paper is to take the first step towards this improvement, by establishing a baseline magnitude of the static metrological factors that describe the precision of measuring the tip location of a pointer using a modern optical mocap system, and to examine how this precision is influenced by the design of the pointer and how it might be improved.* It is hypothesised that the pointer geometry and marker arrangement will have significant influence on the tracking precision of the pointers.

It is worth noting, that accurate positional measurements has a long history in science and metrology. Coordinate measurement machines (CMMs) are specialised instruments whose only focus is to perform precise and accurate measurement of coordinates (ISO 10360 series of standards). Surgical navigation systems used mechanical arms [35] to measure pointer location at first, but switched to optical systems [36, 37] for the added flexibility in the surgical theatre. However, none of these systems have to be able to perform their accurate measurements with the data rate (60–120 Hz measurements of possibly 50–100 points simultaneously) and in a measurement volume (starting from the size of a small room) that a motion capture system has to in order to do dynamic motion analysis studies.

The terms’ accuracy’ and ‘precision’ in this paper are used in accordance with the ISO 5725-1 standard. *Precision* refers to the consistency (how close are the measured values to the mean measured value), and *trueness* refers to how close is the mean of the measurements to the true value. *Accuracy* is the blanket term used for the combination of both *precision* and *trueness*.

## Materials and Methods

### Measurement System

An OptiTrack infrared optical motion capture system was used for the experiments, located in the Motion Laboratory of the Department of Mechatronics, Optics and Mechanical Engineering Informatics at Budapest University of Technology and Economics. The system comprises 18 Flex13 cameras, the Motive:Body software (V2.3.) and 12.7 mm (1/2″) diameter retroreflective markers. The measurement volume is 4 × 2.5 × 3 metres. Tracking data is streamed over the local network to a custom software used for reconstructing the tip position based on the measured rigid body data of the pointer.

With proper calibration and scaling compensation, sufficient measurement trueness is achieved. The root mean square (RMS) of distance errors for determining marker position is sub millimetre (0.76 mm) compared to a geodesic surveying system, and the distance error of two markers over a 4.72 metre distance is < 0.05% compared to a surveying tape measure [38]. Systematic errors affecting trueness in uncompensated mocap systems correlate with the distance from the origin [38, 39] and can be considered constant within a small volume. The calibration of anatomical landmarks is a relative measurement that is performed inside a small volume, where the difference in any present systematic error between the segment and landmark locations would be so minimal that the relative landmark position is virtually unaffected. As a result, trueness of the measured marker positions is assumed, and only precision is of concern.

### Instrumented Pointers

**Fig. 2:**
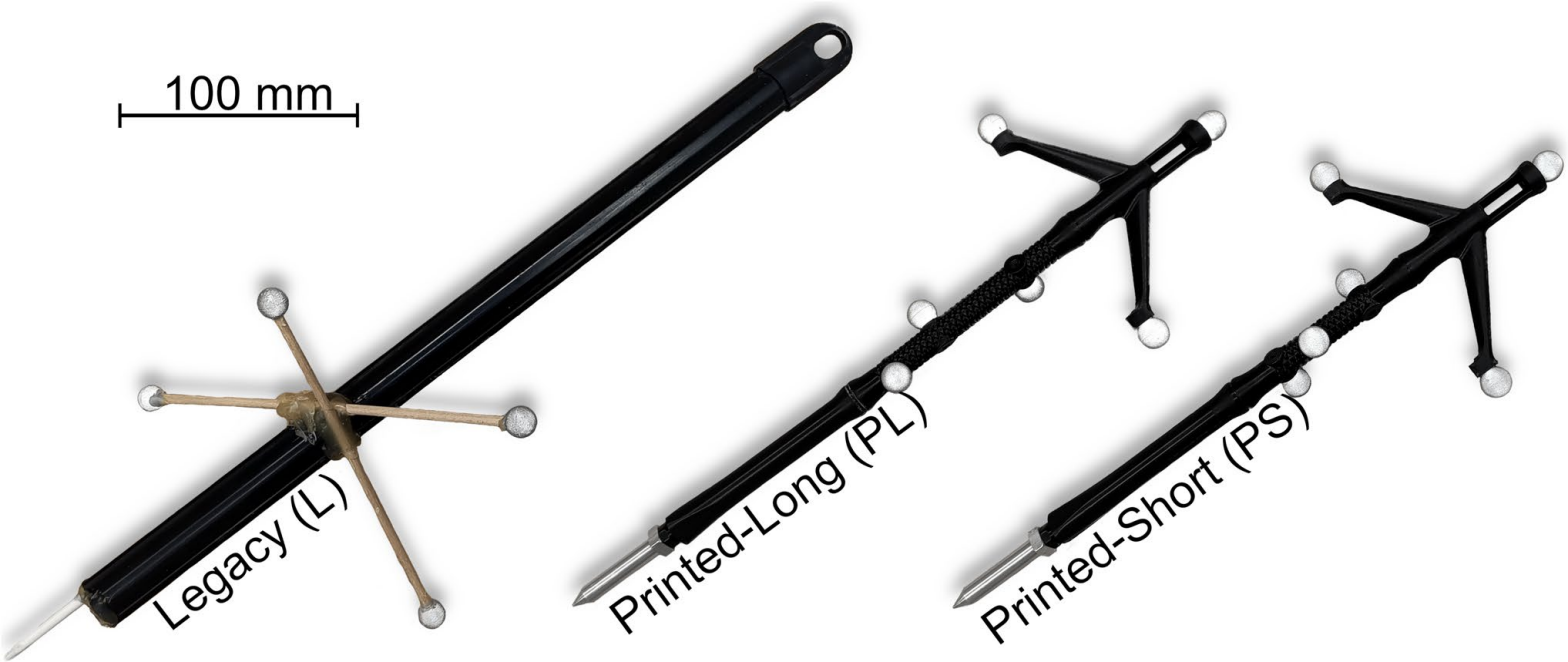
Tested instrumental pointers. The *Legacy* pointer was the designated pointer in the past for motion analysis in the laboratory, constructed from spare plastic parts, wooden sticks and hot glue. The *Printed-Long* and *Printed-Short* pointers were designed and 3D printed for this research. The two designs are identical apart from the middle section of the pointer body, which is 3/4th of the length (28.75 mm shorter) on the short pointer

Three pointer geometries were tested to get an overview of the different precision factors. One pointer was the designated instrument in the past for motion analysis in the laboratory (*Legacy—L*), whilst the other two designs were variations of a pointer specifically designed and 3D printed (*Printed Long—PL* and *Printed Short—PS*) for these experiments (Fig. 2).

The Legacy pointer has four passive markers in a quasi-planar configuration, mounted with wooden skewers and hot glue to the plastic body. The distance between the barycentre of markers and the tip is approximately 150 mm along the pointer’s long axis. The tip was calibrated with two different approaches. For the marker-based method, a marker was placed on the tip and was set to be the pivot point for the corresponding rigid body object in the mocap software. The streamed rigid body origin position now coincided with the tip of the pointer, removing the need for additional calculations by custom software. The functional method determines the tip position based on a short measurement, where the tip is fixed in place, and the pointer body is rotated around it. A more detailed explanation of the different tip calibration methods will be presented in the next instalment of the article series. The Legacy pointer calibrated with the marker-based and functional methods is treated as two different pointers, designated as Legacy-Marker (LM) and Legacy-Functional (LF) going forward.

The new pointers have an FDM 3D printed three-part body made from PLA (Fig. 2*PL* and *PS*) and a machined aluminium tip. The tip and markers are mounted via M4 threads. The geometries of the two printed pointers are identical apart from the middle body segment, which is 3/4^th^ of the length (28.75 mm shorter) on the shorter pointer. The distance between marker barycentre and pointer tip is approximately 245 mm for *PL* and 228 mm for *PS*. The *Printed* pointers were calibrated using the functional approach only, as the tip geometry did not allow the use of the marker-based method.

### Experimental Design

Static metrological factors were established with two measurements. Firstly, the position of a single, stationary marker in the centre of the measurement volume was recorded for a full minute, characterising the instrumental noise of the measurement system. Secondly, each pointer variation (*LM*, *LF*, *PL* and *PS*) was measured for 1000 frames at three locations.

**Fig. 3:**
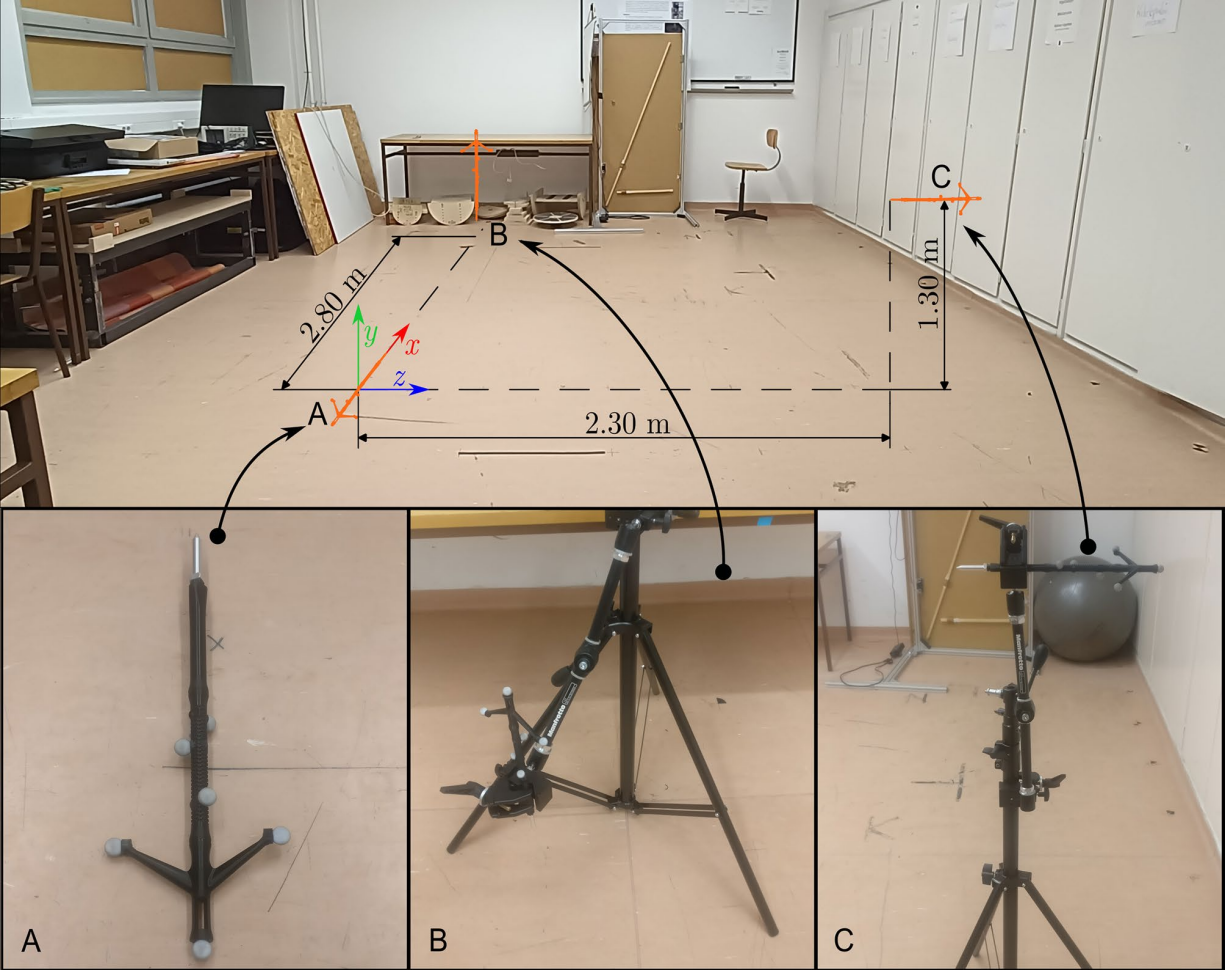
Pointer setup for the static pointer measurements. Orange pointer silhouette indicating approximate pointer placement within the measurement volumes, whilst separate photographs show the actual arrangements at location A, B and C. Origin of the global coordinate system is located at point A, with directions indicated by the coloured axes

- At location A, the pointer was placed on the ground over the origin, with the tip pointing towards the positive X direction (Fig. 3*—Location A*).
- At location B, the pointer was fixed vertically using a camera clamp slightly above ground level, close to the measurement volume edge along the X axis ~ 2.8 metres from the origin, with the tip pointing downwards in the negative Y direction (Fig. 3*—Location B*).
- At location C, the pointer was fixed horizontally ~ 1.3 metres above ground using the camera clamp, close to the measurement volume edge ~ 2.3 metres from the origin along the Z axis, with the tip pointing in the negative Z direction (Fig. 3*—Location C*).

Tip calibration of the pointers was performed, followed by recording the tip position for 1000 frames at each location before moving on to the next pointer.

### Processing and Evaluation

The metric used to describe a single observation is the three-dimensional error ${e}_{3D}$, the Euclidian distance between the observation and the mean location:

1 $$\begin{array}{c}{e}_{3D}=\sqrt{{\left({r}_{x}-{\overline{r} }_{x}\right)}^{2}+{\left({r}_{y}-{\overline{r} }_{y}\right)}^{2}+{\left({r}_{z}-{\overline{r} }_{z}\right)}^{2}}=\sqrt{{{e}_{x}}^{2}+{{e}_{y}}^{2}+{{e}_{z}}^{2}}\end{array}$$

where

- ${r}_{x}{,r}_{y},{r}_{z}$ are the individual coordinates of a measured 3D position vector,
- ${\overline{r} }_{x},{\overline{r} }_{y},{\overline{r} }_{z}$ are the individual coordinates of the mean 3D position vector, and
- ${e}_{x},{e}_{y},{e}_{z}$ are the individual coordinates for ${e}_{3D}$.

The precision of measurements is described with the root mean square error (RMS_e_) and the radius of a 95% confidence sphere (${R}_{95}$). Given ${e}_{3D}$ for a set of $n$ observations, RMS_e_ is defined as:

2 $$\begin{array}{c}{\text{RMS}}_{e}=\sqrt{\frac{1}{n}\sum_{i=1}^{n}{\left({e}_{3D,i}\right)}^{2}}\end{array}$$

The radius ${R}_{95}$ represents a sphere centred on the mean position that is expected to include an observation with 95% probability based on the empirical Cumulative Distribution Function (eCDF):

3 $$\begin{array}{c}{R}_{95}\to P\left({e}_{3D}<{R}_{95}\right)=0.95\end{array}$$

Non-parametric statistical methods were used for hypothesis testing, as the data was not normally distributed. Normality was tested with several methods, including Anderson-Darling, Shapiro-Wilk and visual tests. Individual coordinates were often multimodal as a result of camera flicker (the slight frame-to-frame change in the observed position of a marker as a result of it being very close to the detection threshold for one or more cameras), and ${e}_{3D}$ is by nature not normally distributed. Standard outlier detection based on 1.5 IQR distance was too strict because of the multimodality, but ${e}_{3D}>2 \cdot {R}_{95}$ was found to be a suitable criterion. After removing outliers, all ${e}_{3D}$ and subsequent metrics were recalculated, and outlier detection was performed iteratively until convergence.

For evaluation of the static pointer tests, the measured tip coordinates were transformed from laboratory to relative coordinates aligned with the pointer. Relative coordinates are designated as *axial* (${r}_{a}$), *orthogonal* (${r}_{o}$) and *planar* (${r}_{p}$) coordinates: the *axial* coordinates are in the direction of the long axis of the pointers, *orthogonal* coordinates are orthogonal to the plane of the pointer (towards the viewer for all pointers in Fig. 2), and *planar* coordinates are orthogonal to the previous two, parallel to the plane of the pointer. Levene’s test was used to determine if there is a significant difference in the variance of the relative coordinates (*α* = 0.05) when comparing between the different coordinate directions, the different locations or between the different pointers, with the other factors being identical. Kolmogorov-Smirnov test was used to determine if the ${e}_{3D}$ distribution differ significantly (*α* = 0.05) when comparing between the measurement location for a given pointer or between different pointers at the same measurement location. RMS_*e*_ and ${R}_{95}$ was calculated for every pointer/location measurement set. Python 3.11.4 was used for all data analysis with the pandas (2.0.3), numpy (1.25.1), scipy (1.11.1) and scikit-learn (1.2.2) libraries.

## Results

### Single Marker Precision

**Fig. 4:**
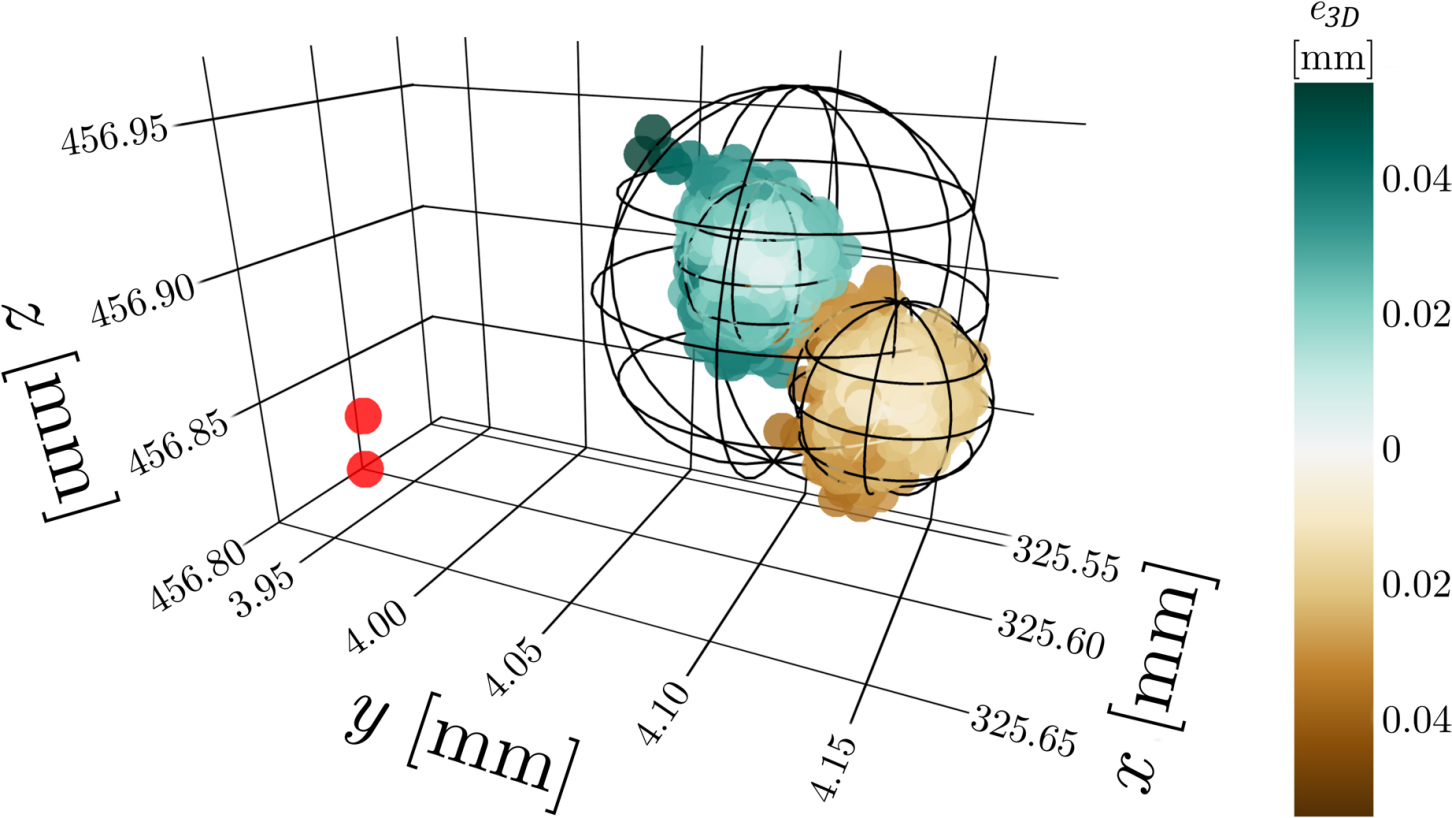
Spatial distribution of measured static marker positions. The different colours indicate the two distinct clusters formed by the measurement positions as a result of camera flicker; colour intensity indicates the ${e}_{3D}$ for a given observation within its own cluster. Red dots indicate outlier observations

In total, 7354 frames were recorded to establish the system’s single marker precision. The spatial distribution of the observations can be seen on Fig. 4. Two outlier points were removed (0.0272%). Due to the camera flicker effect, two distinct clusters of positions are visible in the data. The spectral clustering algorithm of the scikit-learn library was used to cluster the data into two clusters, with 5769 and 1583 observations in each. The RMS_*e*_ and ${R}_{95}$ for the single marker measurement is given in Table 1.

**Table 1:** Precision of the single marker measurement in terms of RMS_e_ and ${R}_{95}$

Cluster	No. of observations	RMS_*e*_ (mm)	${R}_{95}$(mm)
Main cluster (turquoise)	5769	0.013	0.026
Smaller cluster (brown)	1583	0.014	0.027
Combined	7352	0.031	0.060

### Static Pointer Precision

**Fig. 5:**
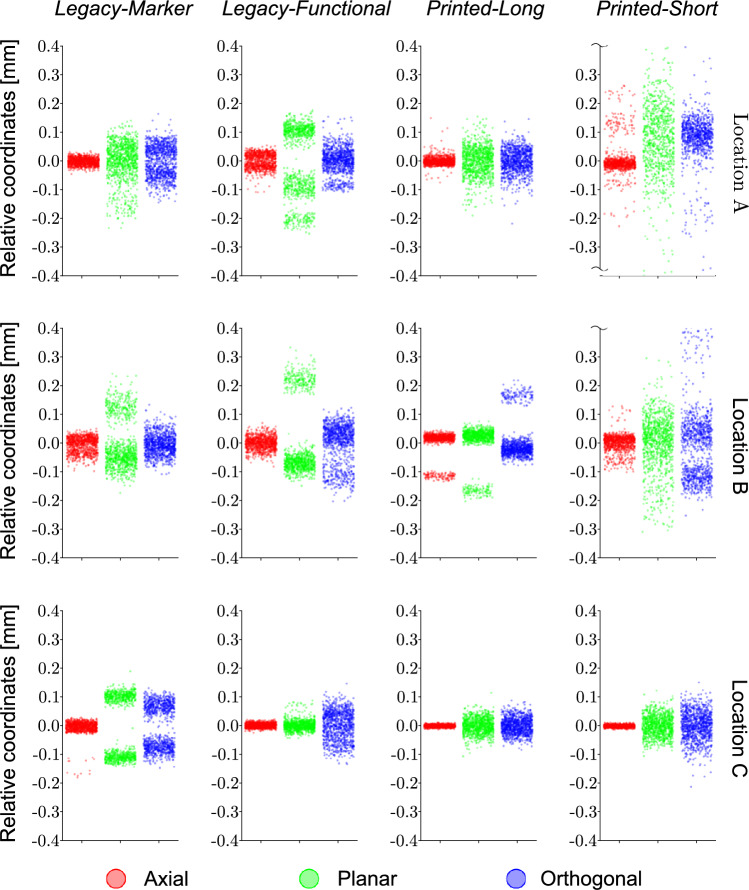
Relative coordinate distribution of tip location of static pointers based on 1000 frames per configuration. Columns of diagrams represent the different pointers whilst rows represent the measurement location. Colours red, green and blue indicate the axial, planar and orthogonal relative coordinates respectively. for the measurement. Broken axis ends at location A and B for the *Printed-Short* pointer indicate observations that fall outside the chosen range of axes. Smaller vertical spread indicates a more precise measurement

Levene’s test showed that practically all factors have a statistically significant influence on static tip precision. Variances between the relative coordinates of a pointer at a given location rejected H_0_ in all but 3 cases out of 36 (*p < 0.01*): between the planar and orthogonal coordinates of *PL* at location B (*p = 0.888*) and C (*p = 0.124*) and of LM at location A (*p = 0.061*). The variance of relative coordinates of a pointer at different measurement locations was significantly different for all cases (*p < 0.01*). Variance of relative coordinates between different pointers at the same location rejected H_0_ (*p < 0.01*) for all but 5 combinations out of 54 (*LF-PL* planar coordinates *p = 0.061* and LM-PL axial coordinates *p = 0.355* at point A, LM-PS axial *p = 0.077* and orthogonal *p = 0.564* coordinates at point B, PL-PS axial coordinates at point C). The different relative coordinates for the pointers at all three locations are visualised on Fig. 5.

Two-sided Kolmogorov-Smirnov tests rejected identical distributions of ${e}_{3D}$ for all cases both between measurement locations for the same pointer and between different pointers at the same location (*p < 0.001* for all cases except for comparing the *Legacy-Functional* pointer with the *Printed Short* one at location C, in which case *p = 0.036*). Resulting RMS_*e*_ and ${R}_{95}$ values are given in Table 2*.*

**Table 2:** RMS_e_ and ${R}_{95}$ for the static pointer tests, given in mm

Pointer	RMS_*e*_ (mm)—${R}_{95}$ (mm)			Mean
	Location			
	A	B	C	
LM	0.086–0.169	0.097–0.172	0.133–0.155	0.105–0.165
LF	0.132–0.228	0.146–0.273	0.053–0.095	0.110–0.199
PL	0.065–0.117	0.103–0.267	0.038–0.069	0.069–0.151
PS	0.539–1.430	0.169–0.396	0.059–0.109	0.256–0.645
Mean	0.205–0.486	0.129–0.277	0.071–0.107	

## Discussion

The aim of the present paper was to establish a baseline magnitude of static metrological factors describing the precision a pointer used with a motion analysis focused modern optical mocap system, and examine how this precision is influenced by the design of the pointer and how it might be improved, if at all. Single marker precision of the mocap system was better than 0.1 mm ${R}_{95}$. Static pointer precision is approximately 0.1 mm RMS_e_ and 0.2 mm ${R}_{95}$, but if pointer markers are located closer than what the mocap system can resolve, larger errors can be expected (*Printed-Short* pointer). Beyond meeting this basic criterion, improvements by optimising pointer geometry are likely possible but very limited.

Single marker precision of the mocap system is satisfactory, with RMS_e_ = 0.031 mm and ${R}_{95}$= 0.060 mm. The measurement did not yield very clean data, Fig. 4 illustrates the issue of camera flicker, as distinct clusters of points are visible from one or more cameras being close to the threshold of detecting the marker. Based on evaluating the clusters separately (Table 1), marker noise could be halved under perfect conditions, but it is in no way guaranteeable throughout the whole measurement volume. This clearly indicates, that a mocap system’s precision is still nowhere close to a dedicated dimensional measurement systems [40–42], although achieving that level of precision would matter little in the face of the human error that gets introduced in CAST measurements.

The static pointer measurements provide insights into how pointer geometry affects precision. Minor but statistically significant differences (Kolmogorov-Smirnov tests, *p < 0.05*) were found between the different pointers regarding the overall precision of the tip location. The longer *Printed* pointer performed the best (0.069 mm RMS_e_ − 0.151 mm ${R}_{95}$ mean precision), even coming close to single marker precision at location C (0.038 mm RMS_e_ − 0.069 mm ${R}_{95}$). Despite having fewer markers (4 vs 6) in an ad hoc arrangement, the *Legacy* pointer’s precision was within 0.05 mm of the *Printed-Long* pointer (0.105/0.110 mm RMS_e_ − 0.165/0.199 mm ${R}_{95}$ mean precision, Table 2)*.* These results show considerable improvements over older examples of static precision in literature, such as the static ‘mean precision’ in Motion Analysis Laboratory tests (MAL test, sometimes also called a ‘spot-check’) [43], ranging from ~ 0.47 mm 3D error [44] to ~ 3.87 mm 3D error [28]. These tests used the classic two-marker pointers, which are much more sensitive to the pivot effect [27], and significantly older mocap technology which also improved in the past 20 + years. Were a MAL test be performed with a modern mocap system, and a pointer design, results are likely to be much closer, if not identical to our findings.

The performance of the shorter *Printed* pointer was far worse compared to the other tested instruments, with more than twice the errors (0.256 mm RMS_e_ − 0.645 mm ${R}_{95}$ mean precision). The cause of the problem is not obvious based on the data but was very apparent during the measurements. The markers were located too close together and the mocap system could not properly distinguish them, leading to false marker locations and incorrect pointer measurements.

Pointer geometry and marker arrangement are important factors in pointer precision. Future designs need to maintain sufficient marker spacing, but exact criteria will depend on the properties of the measurement system, such as marker size and the distance of the cameras from their target. Beyond this, only very small improvements can be expected from the optimisation of geometry in the range of a couple hundredths of a mm. As hypothesised, pointer geometry and marker arrangement can have significant effects on static precision. However, it is reasonable to say that at its current level, this factor is not a bottleneck for pointer precision, as the static precision of a well-built pointer is better than the precision with which a human examiner could mark a point even with a perfectly accurate instrument.

Several additional observations can be made based on the data and statistical tests. Firstly, the axial variance of tip location is significantly lower than in the planar or orthogonal directions (Fig. 5). This is to be expected, as the pivot effect has minimal influence on the axial direction compared to the others (Fig. 1). Secondly, camera flicker affects tip position too. However, it seems more common in the *Legacy* pointer than in the *Printed* ones, suggesting the higher number of markers might make the pointer more robust against it. Marker number needs to be balanced with available space to ensure good marker separation and avoid the issues seen with the *Printed-Short* pointer but keep the length short to minimise the pivot effect. Lastly, precision is statistically significantly affected by the location in the measurement volume but not enough to be of practical significance for a well performing pointer.

The limitations of the present study are related to its exploratory nature. The tested pointer geometries are only a small sample of the possible variations, and better geometries might exist. Computer simulation and evolutionary algorithms could be used to find an optimal marker arrangement, but the possible improvements are limited. Single marker performance was only characterised in a single point, and results might vary for the rest of the measurement volume. A more thorough characterisation with a grid of points could help better understand the capabilities of the mocap system and explain some of the variations in the results of the static pointer measurements. However, it is unlikely to affect the conclusions since single marker measurement noise is minimal compared to the other errors. The locations of the static pointer trials were not controlled precisely, and different noise might affect the individual measurements. The repeatability of the measurements is not guaranteed this way, but variations should be minimal and primarily random in a well calibrated mocap system [38, 39].

It is reasonable to assume, that any modern mocap system can operate with satisfyingly low levels of measurement noise, but it’s still good practice to confirm if it is operating optimally. In case the mocap system does not provide self-evaluation after the camera system calibration (which is usually recommended by the manufacturer to be performed daily, prior to measurements, with a warmed-up camera system), users can perform simple measurements similar to the ones presented in this paper, or an adaptation of the spot-check/MAL test [43] mentioned previously.

To conclude, we have examined the static factors of instrumented pointer precision, as the first step in a series of studies to fully characterise, and ultimately optimize the performance of said instrumented pointer in the context of motion capture systems. This paper is the first instalment in a series of studies that aims to do that, and examined the static factors of this precision. The next part will focus on the dynamic precision of the same pointers, to give a full breakdown how precisely a user can mark a desired location using the pointer. This will be the sum of the errors from measuring the pointer tip with the mocap system (static precision), the systematic error between the physical and measured pointer tip dependent on the goodness of tip calibration (tip calibration error), and the precision with which the examiner can place the physical tip at the desired point (marking error). Once the full extent of the different components of precision are know, we will be able to focus on optimizing the pointer geometry and any calibration approach in order to minimize errors, helping to achieve consistent and accurate anatomical landmark calibrations.

## References

[1] Cappozzo, A., U. Della Croce, A. Leardini, and L. Chiari. Human movement analysis using stereophotogrammetry Part 1: theoretical backgrond. *Gait Posture*. 2005. 10.1016/j.gaitpost.2004.01.010. 15639398 10.1016/j.gaitpost.2004.01.010

[2] Tajino, J., A. Ito, M. Tanima, S. Yamaguchi, H. Iijima, A. Nakahata, et al. Three-dimensional motion analysis for comprehensive understanding of gait characteristics after sciatic nerve lesion in rodents. *Sci. Rep.* 2018. 10.1038/s41598-018-31579-z. 30206259 10.1038/s41598-018-31579-z PMC6133925

[3] Herbst, Y., L. Zelnik-Manor, and A. Wolf. Analysis of subject specific grasping patterns. *PLoS ONE*. 2020. 10.1371/journal.pone.0234969. 32640003 10.1371/journal.pone.0234969 PMC7343174

[4] Mendez-Angulo, J. L., A. M. Firshman, D. M. Groschen, P. J. Kieffer, and T. N. Trumble. Impact of walking surface on the range of motion of equine distal limb joints for rehabilitation purposes. *Vet. J.* 2014. 10.1016/j.tvjl.2013.12.001. 24556081 10.1016/j.tvjl.2013.12.001

[5] Ross, G. B., B. Dowling, N. F. Troje, S. L. Fischer, and R. B. Graham. Objectively differentiating movement patterns between elite and novice athletes. *Med. Sci. Sport Exerc.* 2018. 10.1249/MSS.0000000000001571. 10.1249/MSS.0000000000001571 29420437

[6] Petró, B., and R. M. Kiss. Validation of the estimated torques of an open-chain kinematic model of the human body. *Period Polytech. Mech. Eng.* 2022. 10.3311/PPme.19920.

[7] Cao, S., and R. Nevatia. Forecasting human pose and motion with multibody dynamic model. *IEEE Winter Conf. Appl. Comput. Vis.* 2015:191–198. 10.1109/WACV.2015.33.

[8] Hwang, S. J., H. S. Choi, and Y. H. Kim. Motion analysis based on a multi-segment foot model in normal walking. *Annu. Int. Conf. IEEE Eng. Med. Biol. Soc.* 4:5104–5106, 2004. 10.1109/IEMBS.2004.1404410. 10.1109/IEMBS.2004.1404410 17271466

[9] Collins, T. D., S. N. Ghoussayni, D. J. Ewins, and J. A. Kent. A six degrees-of-freedom marker set for gait analysis: repeatability and comparison with a modified Helen Hayes set. *Gait Posture*. 2009. 10.1016/j.gaitpost.2009.04.004. 19473844 10.1016/j.gaitpost.2009.04.004

[10] Grood, E. S., and W. J. Suntay. A joint coordinate system for the clinical description of three-dimensional motions: application to the knee. *J. Biomech. Eng.* 1983. 10.1115/1.3138397. 6865355 10.1115/1.3138397

[11] Delp, S. L., F. C. Anderson, A. S. Arnold, P. Loan, A. Habib, C. T. John, et al. OpenSim: open source to create and analyze dynamic simulations of movement. *IEEE Trans. Biomed. Eng.* 2007. 10.1109/TBME.2007.901024. 18018689 10.1109/TBME.2007.901024

[12] Camomilla, V., A. Cappozzo, and G. Vannozzi. Three-dimensional reconstruction of the human skeleton in motion. In: Handbook of human motion, Cham: Springer, 2018, pp. 17–45.

[13] Cappozzo, A., F. Catani, U. Della Croce, and A. Leardini. Position and orientation in space of bones during movement: anatomical frame definition and determination. *Clin. Biomech.* 10:171, 1995. 10.1016/0268-0033(95)91394-t 11415549

[14] Camomilla, V., R. Dumas, and A. Cappozzo. Human movement analysis: the soft tissue artefact issue. *J. Biomech.* 2017. 10.1016/j.jbiomech.2017.09.001. 28923393 10.1016/j.jbiomech.2017.09.001

[15] Manal, K., I. McClay, S. Stanhope, J. Richards, and B. Galinat. Comparison of surface mounted markers and attachment methods in estimating tibial rotations during walking: an in vivo study. *Gait Posture*. 2000. 10.1016/S0966-6362(99)00042-9. 10664484 10.1016/s0966-6362(99)00042-9

[16] Alexander, E. J., and T. P. Andriacchi. Correcting for deformation in skin-based marker systems. *J. Biomech.* 34:355, 2001. 11182127 10.1016/s0021-9290(00)00192-5

[17] Schallig, W., G. J. Streekstra, C. M. Hulshof, R. P. Kleipool, J. G. G. Dobbe, M. Maas, et al. The influence of soft tissue artifacts on multi-segment foot kinematics. *J. Biomech.* 2021. 10.1016/j.jbiomech.2021.110359. 33730563 10.1016/j.jbiomech.2021.110359

[18] Lahkar, B. K., P.-Y. Rohan, A. Assi, H. Pillet, X. Bonnet, P. Thoreux, et al. Development and evaluation of a new methodology for soft tissue artifact compensation in the lower limb. *J. Biomech.* 2021. 10.1016/j.jbiomech.2021.110464. 33932915 10.1016/j.jbiomech.2021.110464

[19] Ancillao, A., E. Aertbeliën, and J. De Schutter. Effect of the soft tissue artifact on marker measurements and on the calculation of the helical axis of the knee during a gait cycle: a study on the CAMS-Knee data set. *Hum. Mov. Sci.* 2021. 10.1016/j.humov.2021.102866. 34509901 10.1016/j.humov.2021.102866 PMC8631460

[20] Fiorentino, N. M., P. R. Atkins, M. J. Kutschke, K. Bo Foreman, and A. E. Anderson. Soft tissue artifact causes underestimation of hip joint kinematics and kinetics in a rigid-body musculoskeletal model. *J. Biomech.* 2020. 10.1016/j.jbiomech.2020.109890. 32636003 10.1016/j.jbiomech.2020.109890 PMC7405358

[21] Rácz K, Nagymáté G, Kiss RM. Effect of anatomical landmark placement variation on the angular parameters of the lower extremities. Proc. 13th IASTED Int. Conf. Biomed. Eng. BioMed 2017, 2017, p. 158–63. 10.2316/P.2017.852-037.

[22] Piazza, S. J., and P. R. Cavanagh. Measurement of the screw-home motion of the knee is sensitive to errors in axis alignment. *J. Biomech.* 2000. 10.1016/S0021-9290(00)00056-7. 10828334 10.1016/s0021-9290(00)00056-7

[23] Morton, N. A., L. P. Maletsky, S. Pal, and P. J. Laz. effect of variability in anatomical landmark location on knee kinematic description. *J. Orthop. Res.* 2007. 10.1002/jor. 17506082 10.1002/jor.20396

[24] Camomilla, V., A. Cereatti, A. G. Cutti, S. Fantozzi, R. Stagni, and G. Vannozzi. Methodological factors affecting joint moments estimation in clinical gait analysis: a systematic review. *Biomed. Eng.* 2017. 10.1186/s12938-017-0396-x. 10.1186/s12938-017-0396-x PMC5563001 28821242

[25] Della Croce, U., A. Leardini, L. Chiari, and A. Cappozzo. Human movement analysis using stereophotogrammetry Part 4: assessment of anatomical landmark misplacement and its effects on joint kinematics. *Gait Posture*. 2005. 10.1016/j.gaitpost.2004.05.003. 15639401 10.1016/j.gaitpost.2004.05.003

[26] Chiari, L., C. U. Della, A. Leardini, and A. Cappozzo. Human movement analysis using stereophotogrammetry Part 2: instrumental errors. *Gait Posture*. 2005. 10.1016/j.gaitpost.2004.04.004. 15639399 10.1016/j.gaitpost.2004.04.004

[27] Erdemir A, Piazza SJ. Simulation-based design of a pointer for accurate determination of anatomical landmarks. Proc. 23rd Annu. Meet. Am. Soc. Biomech., Pittsburgh, PA: 1999, p. 286–7.

[28] Della Croce, U., A. Cappozzo, and D. Kerrigan. Pelvis and lower limb anatomical landmark calibration precision and its propagation to bone geometry and joint angles. *Med. Biol. Eng. Comput.* 1999. 10.1007/BF02513282. 10396818 10.1007/BF02513282

[29] Rácz, K., G. Nagymáté, T. Kovács, T. Bodzay, and R. M. Kiss. Accuracy of anatomical landmark placement methods for gait analysis. *Int. J. Mech. Control*. 19:19, 2018.

[30] Rabuffetti, M., G. Baroni, M. Ferrarin, G. Ferrigno, and A. Pedotti. Self-marking of anatomical landmarks for on-orbit experimental motion analysis compared to expert direct-marking. *Hum. Mov. Sci.* 21:439, 2002. 12450678 10.1016/s0167-9457(02)00115-x

[31] Christoforetti, J., J. De Long, B. T. Hanypsiak, M. Suri, B. G. Domb, J. C. Snibbe, et al. Precision and accuracy of identification of anatomical surface landmarks amongst 30 expert hip arthroscopists. *Am. J. Orthop.* 29:e168, 2017. 28235126

[32] Van Sint, Jan S., and C. U. Della. Identifying the location of human skeletal landmarks: why standardized definitions are necessary—a proposal. *Clin. Biomech.* 2005. 10.1016/j.clinbiomech.2005.02.002. 10.1016/j.clinbiomech.2005.02.002 15927740

[33] Van Sint Jan S. Color atlas of skeletal landmark definitions: guidelines for reproducible manual and virtual palpations. 1st edition. Churchill Livingstone; 2007.

[34] Tawy, G. F., and P. Rowe. Is the instrumented-pointer method of calibrating anatomical landmarks in 3D motion analysis reliable? *J. Biomech.* 2017. 10.1016/j.jbiomech.2017.01.019. 28143654 10.1016/j.jbiomech.2017.01.019

[35] Golfinos, J. G., B. C. Fitzpatrick, L. R. Smith, and R. F. Spetzler. Clinical use of a frameless stereotactic arm: results of 325 cases. *J. Neurosurg.* 1995. 10.3171/jns.1995.83.2.0197. 7616261 10.3171/jns.1995.83.2.0197

[36] Colchester, A. C. F., J. Zhao, K. S. Holton-Tainter, C. J. Henri, N. Maitland, P. T. H. Roberts, et al. Development and preliminary evaluation of VISLAN, a surgical planning and guidance system using intra-operative video imaging. *Med. Image Anal.* 1996. 10.1016/S1361-8415(01)80006-2. 9873922 10.1016/s1361-8415(01)80006-2

[37] Rohling, R., P. Munger, J. M. Hollerbach, and T. Peters. Comparison of relative accuracy between a mechanical and an optical position tracker for image-guided neurosurgery. *Comput. Aided Surg.* 1995. 10.3109/10929089509106823. 10.1002/(SICI)1522-712X(1995)1:1<30::AID-IGS5>3.0.CO;2-N 9079424

[38] Nagymáté, G., T. Tuchband, and R. M. Kiss. A novel validation and calibration method for motion capture systems based on micro-triangulation. *J. Biomech.* 2018. 10.1016/j.jbiomech.2018.04.009. 29678420 10.1016/j.jbiomech.2018.04.009

[39] Nagymáté, G., and R. M. Kiss. Motion capture system validation with surveying techniques. *Mater. Today Proc.* 2018. 10.1016/j.matpr.2018.08.107.

[40] Kovač, I., and A. Frank. Testing and calibration of coordinate measuring arms. *Precis. Eng.* 2001. 10.1016/S0141-6359(00)00057-X.

[41] Küng, A., F. Meli, and R. Thalmann. Ultraprecision micro-CMM using a low force 3D touch probe. *Meas. Sci. Technol.* 2007. 10.1088/0957-0233/18/2/S01.

[42] Vermeulen, M. M. P. A., P. C. J. N. Rosielle, and P. H. J. Schellekens. Design of a high-precision 3D-coordinate measuring machine. *CIRP Ann. Manuf. Technol.* 1998. 10.1016/s0007-8506(07)62871-6.

[43] Della Croce, U., and A. Cappozzo. A spot check for estimating stereophotogrammetric errors. *Med. Biol. Eng. Comput.* 2000. 10.1007/BF02347045. 10912341 10.1007/BF02347045

[44] Cappozzo, A., F. Catani, A. Leardini, M. G. M. Benedetti, and C. U. Della. Position and orientation in space of bones during movement: experimental artefacts. *Clin. Biomech.* 1996. 10.1016/0268-0033(95)00046-1. 10.1016/0268-0033(95)00046-1 11415604

